# Primary Spontaneous Pneumothorax: Results of Surgical Treatment and Analysis of Risk Factors for Post-Operative Recurrence—A Retrospective Cohort Analysis

**DOI:** 10.3390/jcm15072557

**Published:** 2026-03-27

**Authors:** Serena Zanardo, Francesco Londero, Yvonne Beorchia, Luigi Castriotta, Elisa De Franceschi, William Grossi, Gianluca Masullo, Andrea Zuin

**Affiliations:** 1 Department of Cardiac, Thoracic, Vascular Sciences and Public Health, University of Padua, 35128 Padua, Italy; serena.zanardo@aopd.veneto.it; 2 Department of Thoracic Surgery, Santa Maria della Misericordia University Hospital, 33100 Udine, Italy; elisa.defranceschi@asufc.sanita.fvg.it (E.D.F.); william.grossi@asufc.sanita.fvg.it (W.G.); gianluca.masullo@asufc.sanita.fvg.it (G.M.); andrea.zuin@asufc.sanita.fvg.it (A.Z.); 3 Institute of Hygiene and Evaluative Epidemiology, Santa Maria della Misericordia University Hospital, 33100 Udine, Italy; yvonne.beorchia@gmail.com (Y.B.); luigi.castriotta@asufc.sanita.fvg.it (L.C.)

**Keywords:** pneumothorax, VATS, thoracic surgery, lung, pleurodesis, spontaneous pneumothorax

## Abstract

**Background/Objectives**: Several studies previously investigated the risk factors for post-operative recurrence of primary spontaneous pneumothorax (PSP), with conflicting results. Identification of patients at greater risk of recurrence may help optimize therapeutic strategies. The aim of this study is to identify potential predictors of post-operative recurrence of PSP and compare our results with the available literature. **Methods**: We retrospectively evaluated all patients who underwent surgery for PSP at our institution between January 2005 and December 2022. We analyzed data on patient characteristics, surgical details, method of pleurodesis and perioperative outcomes and used Cox regression analysis to identify predictors post-operative ipsilateral recurrence. **Results**: 226 patients were included in our study, of which 29 (12.8%) developed an ipsilateral recurrence of pneumothorax. A positive history of previous contralateral episodes (37.9% vs. 19.3%, *p* = 0.03) and the positioning of larger chest drains after the procedure (65.5% vs. 44.8%, *p* = 0.032) were more frequent in the recurrence group. At multivariable regression analysis, a history of previous contralateral pneumothorax was found to be the only independent predictive factor of pneumothorax recurrence (HR 2.16, 95% C.I. 1.001–4.662, *p* = 0.049). **Conclusions**: Previous contralateral pneumothorax is a risk factor for the development of post-operative recurrences.

## 1. Introduction

Primary spontaneous pneumothorax (PSP) is a relatively common condition occurring in patients without any underlying lung morbid condition [1,2]. It mostly affects young, tall and thin males with an incidence of approximately 7.4–18 and 1.2–6 cases per 100,000 people per year for men and women respectively, with a peak incidence in the second or third decade of age [3].

The high incidence of recurrences represents one of the primary issues of this condition, causing an increase in hospitalization rates, which in turn is an important factor of morbidity and increased healthcare expenditures [4,5,6,7].

According to the main guidelines [1,8], the treatment of PSP depends on various clinical and radiographic factors and includes observation, oxygen administration, simple aspiration or chest drainage [9,10]. However, chest tube thoracostomy alone is associated with a high recurrence rate [4]. In case of recurrence or failure of conservative management, surgical treatment is advised and proved effective in decreasing the rate of PSP recurrence (0–13% versus 22.8–42% with only thoracostomy) [11,12].

Surgery is conventionally performed via Video-Assisted Thoracic Surgery (VATS) and reckons on the resection of lung bullae when present and pleurodesis, which consists of obliterating the pleural space and preventing recurrence of pneumothorax (PNX). There are various means to obtain pleurodesis during surgery, such as intrapleural instillation of chemical agents, parietal pleurectomy or pleural abrasion. Several approaches have been described in the literature, each with potential advantages and limitations. Although no universal consensus has been reached regarding the most effective strategy, the choice of technique is often guided by surgeon preference, institutional experience, and the specific clinical context. However, surgery does not exclude the possibility of further recurrences.

Recent evidence from large cohort studies and systematic reviews has further clarified recurrence patterns after different management strategies for PSP [13,14]. Recurrence rates following conservative treatment remain relatively high, with reported rates ranging from approximately 20% to over 50% after a first episode, most events occurring within the first year [13,15]. In contrast, surgical management has been associated with significantly lower recurrence rates, generally reported around 5–15% in contemporary series. Nevertheless, post-operative recurrence still occurs in a non-negligible proportion of patients [13]. Identifying patients at increased risk of relapse can help optimize treatment strategies: the risk of PSP recurrence after surgery has been analyzed by various studies, albeit with conflicting results and remarkable heterogeneity. Therefore, the aim of this study is to identify potential risk factors for ipsilateral post-operative recurrence of PSP and contextualize our results with those of the available literature.

## 2. Materials and Methods

This paper was structured according to the Strengthening the Reporting of Observational Studies in Epidemiology (STROBE) statement [16]. The study was approved by the local ethical committee (Comitato Etico Unico Regionale del Friuli Venezia Giulia, CEUR-2024-Os-27). Informed consent for this study was waived by the Ethical Committee due to the retrospective nature of its design.

### 2.1. Population of Study

We performed a retrospective revision of all clinical records of all consecutive patients who underwent surgery for PNX between January 2005 and December 2022 in a single center (S. Maria della Misericordia University Hospital, Udine, Italy).

Inclusion criteria for the study were (1) patients undergoing first surgery for PSP, (2) bullectomy and/or pleurodesis under general anesthesia, (3) post-operative follow-up of at least 6 months, and (4) patients who have already granted consent to have their data processed for research reasons. Exclusion criteria were (1) patients with secondary PNX (secondary spontaneous (SSP), catamenial, iatrogenic, traumatic), and (2) patients submitted to simple chest drainage without pleurodesis and/or bullectomy. The primary endpoint of the study was ipsilateral post-operative recurrence of pneumothorax after surgical treatment for PSP. No secondary endpoints were predefined or specifically analyzed, as the analysis was focused on identifying factors potentially associated with post-operative recurrence.

We collected data on patients’ features (age, sex gender, height, body mass index (BMI), comorbidities), previous episodes of PSP, surgical modalities, and indications for surgery (approach, kind of resection, kind of pleurodesis), post-operative outcomes, and follow-up. In accordance with the guidelines of the Italian Society of Thoracic Surgery [17], surgical treatment of PSP was proposed in the following cases: (1) PSP ipsilateral to the previous episode, (2) PSP contralateral to the previous episode, (3) evidence of air leak following chest drainage lasting for more than 5 days with inability to wean from the drainage tube, (4) patient’s will, (5) PSP associated with hemothorax with hemodynamic instability (emergent indication).

Anthropometric data (weight and height) have been expressed in terms of BMI, in order to standardize the measurements. PNX extension at the time of presentation was measured on chest X-ray (CXR) both at the apex, according to American guidelines [17], and at the lung hilum, according to the British guidelines [18]. Due to the extreme variability of these values and the impossibility of defining measurements in cases of massive PNX, these data have been expressed as categorical variables according to their extent (dimension at the apex: ≤3 cm; 3–6 cm; >6 cm; dimension at the hilum: ≤2 cm; 2–4 cm; >4 cm).

Pleurodesis was recorded using two distinct variables: one specifying whether the patient underwent medical pleurodesis (instillation of talc or other substances) and the other indicating whether the patient underwent surgical pleurodesis (pleurectomy, scarification, or abrasion).

### 2.2. Surgical Technique

Patients were administered general anesthesia and intubated with a double lumen tube or bronchial blocker, in order to ensure lung exclusion of the operated side. Patients were therefore positioned in contralateral decubitus. Surgery was conducted through a VATS approach by creating 1–3 incisions for the introduction of the camera and operating instruments, or through anterolateral or posterolateral thoracotomy.

The identification of air leak, subpleural blebs, or pulmonary dystrophy was accomplished through accurate lung exploration and/or water-seal test. Once identified, these were subjected to either wedge resection, which represented the standard approach in our institution, or ligation. The latter was performed at the discretion of the operating surgeon in selected cases, particularly when small blebs were identified or when anatomical conditions made stapled resection less suitable. During the study period the following staplers were used: Endo GIA^TM^: 60 mm purple cartridges (Medtronic S.p.A, Milan, Italy), Echelon^TM^: 45 mm or 60 mm green or blue cartridges (Ethicon J&J MedTech, Cincinnati, OH, USA), and Signia^TM^: 45 mm or 60 mm purple cartridges (Medtronic S.p.A, Milan, Italy)

Blebs ligation was performed with a pre-shaped loop suture, with no parenchymal tissue removal, while the resection was performed with mechanical staplers. In addition, a pleurodesis technique could have been associated. Mechanical pleurodesis by pleural abrasion represented the most frequently adopted technique. In selected patients, particularly when a higher risk of recurrence was perceived, a more extensive pleural procedure such as partial pleurectomy was performed. Although the overall surgical principles remained consistent throughout the study period—namely treatment of identifiable bullae and induction of pleurodesis—the specific technical choices were not strictly protocolized and could vary according to intraoperative findings and individual surgeon preference.

One or two chest tubes were routinely positioned at the end of the surgical procedure. Wound closure was performed after lung re-expansion under vision and no evidence of further air leaks.

### 2.3. Post-Operative Management

Following extubation, patients were transferred to the surgical ward with vital signs monitoring for the first 24 h. Chest drains were routinely posed on continuous suction for at least 24 h and a CXR was performed on the day of the surgical procedure to confirm lung expansion and absence of early complications (e.g., hemothorax). Drains were posed off suction in the first post-operative day in case of the absence of visible air losses in the drainage system or according to clinical judgment by the operating surgeon. The drain was eventually removed after at least 48 h of absence of visible air losses and if good lung expansion was shown on CXR performed 12 h after drain tube clamping. The patient was discharged if CXR performed 24 h after drain removal confirmed stable lung expansion, excluding the reappearance of the pneumothorax due to the persistence of a parenchymal air fistula.

### 2.4. Follow-Up

Follow-up data were collected through hospital records review and direct telephone contact with patients.

During the follow-up process, five patients were lost to follow-up despite attempts at contact and were therefore excluded from the long-term recurrence assessment.

### 2.5. Post-Operative Recurrence of PSP

Post-operative recurrence was defined as the development of pneumothorax ipsilateral to the side of surgery after post-operative day 31, diagnosed with a chest radiography or chest computed tomography (CT).

Post-operative recurrence was defined as pneumothorax occurring after post-operative day 31 in order to distinguish true recurrence from early post-operative events [18,19]. The first post-operative days are generally considered the standard time frame for reporting surgical morbidity, and events occurring during this period may reflect complications related to the surgical procedure, such as prolonged air leak, incomplete pleurodesis, or staple-line failure rather than a true recurrence of the underlying disease [18,20]. Previous studies on surgery for primary spontaneous pneumothorax have similarly differentiated post-operative pneumothorax from late recurrence using a temporal threshold, commonly around 30 days, as early events may be related post-operative healing processes rather than new disease occurrence [18,19,20].

### 2.6. Statistical Analysis

Categorical data were described as number and percentages, while continuous data were expressed as mean ± standard deviation (sd) or median and interquartile range (IQR), as appropriate. Normality of distributions was assessed with the Shapiro–Wilk test. Comparisons were carried out with Chi-square or Fisher’s exact test for categorical variables and Student’ t or Wilcoxon–Mann–Whitney test for continuous data, as appropriate. Kaplan–Meyer curves were used to depict recurrence-free intervals, and between-group comparisons were evaluated with a log-rank test of equality. A Cox proportional hazards regression analysis was conducted to identify factors associated with PNX recurrence. Variables selection for regression analysis was based on their hypothesized clinical relevance, on their apparent significance in inter-groups comparisons and on their value in previous publications. Variables with a *p*-value < 0.1 at univariate analysis were selected for multivariable regression test. The proportional hazards assumption test was based on Schoenfeld residuals. Statistical significance was set at α < 0.05. All analyses were performed with SAS Enterprise Guide 7.1 (SAS Institute Inc., Cary, NC, USA) software.

## 3. Results

**Figure 1:**
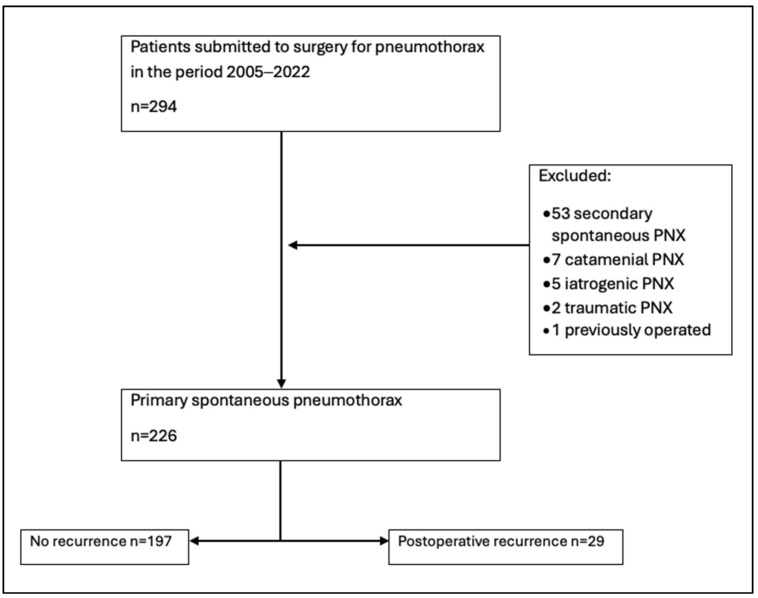
Selection process for study population.

In the considered interval of time 294 patients underwent surgery for pneumothorax and 68 of them were excluded for not meeting the inclusion criteria (Figure 1).

The characteristics of the 226 patients representing the study population are reported in Table 1. The median age of the population was 22 years (IQR 18–30), with a median BMI of 19.8 kg/m^2^ (IQR 18.4–22.2), predominantly composed of male patients (80.5%). A smoking history was present in 102 patients (45.1%) and comorbidities in 62 patients (27.4%). Lung comorbidities were present in 14 patients (22.6%), represented in all cases by allergic asthma.

In most cases, the right hemithorax was affected (58%, Table 2).

No differences were encountered in the proportion of patients who had previous episodes of PNX (recurrence group 82.8% vs. no recurrence group 66.0%, *p* = 0.08) or ipsilateral previous episodes (62.1% vs. 53.8%, *p* = 0.43), whereas previous contralateral episodes were more frequent in the recurrence group (37.9% vs. 19.3%, *p* = 0.03).

Operative data are illustrated in Table 3.

Recurrence of pneumothorax ipsilateral to the previous episode was the main indication to surgery (51.8%). No differences were encountered between the two groups with regard to the reason for surgery. VATS approach was employed in nearly all cases (97.3%). Bullae resection was carried out in 197 patients (87.2%) and pleurodesis in 224 patients (99.1%). A significant difference was observed in the proportion of patients with larger chest drain positioned at the end of intervention (recurrence group vs. no recurrence group (44.8% vs. 65.5%, *p* = 0.032).

**Figure 2:**
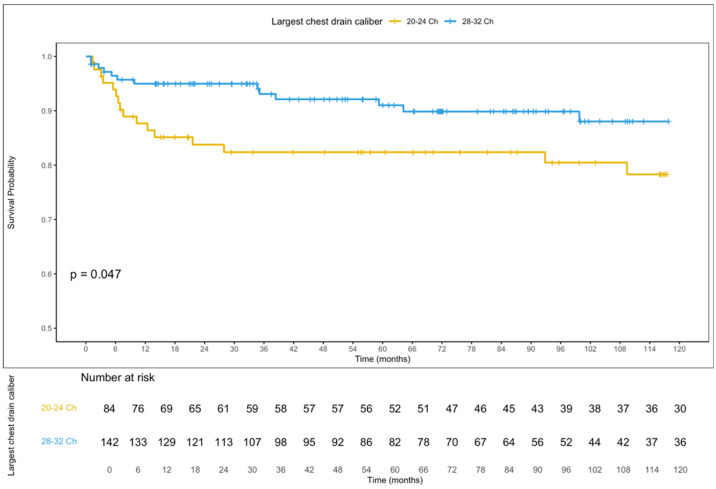
Kaplan–Meier curves illustrating PNX recurrence-free survival stratified by the caliber of the chest drain placed at the end of surgery.

Mean follow-up was 96.65 months (range 6.35–228.59). During the follow-up period, PNX recurrence occurred in 19.0% of patients with smaller chest drains and in 9% of those with larger drains, with a statistically significant difference in late recurrence-free survival (log-rank test, *p* = 0.047, Figure 2). At 10 years, recurrence-free survival was 78.3% (95% C.I., 69.2–88.6) for smaller chest drains and 88.0% (95% C.I., 81.7–94.8) for larger chest drains.

Table 4 resumes post-operative results. On post-operative day 1, CXR revealed an apical room in 119 patients (52.7%) with a median size of 1 cm (IQR 0.5–2). The presence of post-operative air leakage was described in 111 patients (49.1%), which could have contributed to lengthening drains removal time, whose median value was 4 (IQR 4–6) days. Moreover, CXR performed after drains removal showed an apical room in 144 cases (63.7%). Fifty-four patients (23.9%) experienced post-operative complications, consisting mainly of persistent air leaks (11.9%), fever (5.3%), and bleeding (2.3%). No differences were found in the incidence of post-operative complications between the two groups (*p* = 0.643).

During the follow-up period, 29 patients (12.8%) developed an ipsilateral recurrence of pneumothorax after a median time of 89.6 months (IQR 46.2–152). Recurrences were treated conservatively in 19 cases (65.5%), with tube thoracostomy in four patients (13.8%), whereas surgical exploration was deemed necessary in 6 cases (20.7%).

**Figure 3:**
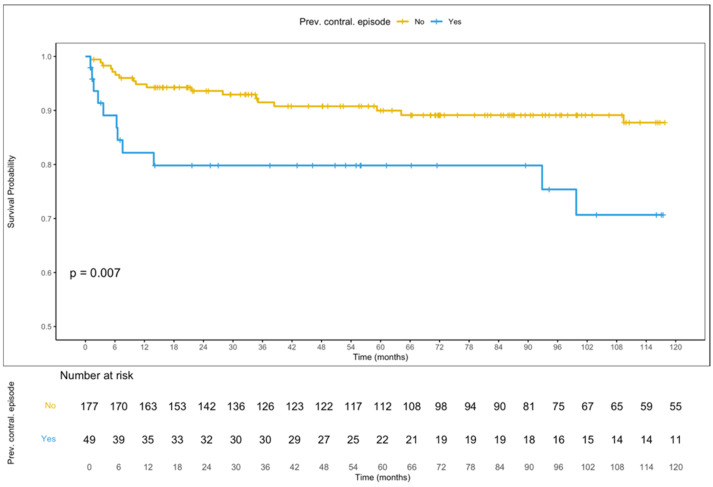
Kaplan–Meier curves illustrating recurrence-free survival of PNX, stratified by history of previous contralateral episodes.

Kaplan–Meier survival analysis (Figure 3) showed a statistically significant difference in survival free from late recurrence between patients with and without a history of previous contralateral PNX (Log-rank *p* = 0.007), with patients having a prior contralateral PNX showing lower survival free from recurrence. At 10 years, recurrence-free survival was 70.7% (95% C.I., 56.5–88.5) in patients who had a previous contralateral episode and 87.8% (95% C.I., 82.4–93.5) in those who did not.

By entering the variables in the Cox regression model, we obtained the results shown below (Table 5). At univariate analysis, BMI (HR 0.87, 95% C.I. 0.748–0.999, *p* = 0.049), history of previous contralateral episode (HR 2.68, 95% C.I. 1.266–5.680, *p* = 0.010) and larger size of the drain positioned at the end of surgery (HR 0.48, 95% C.I. 0.232–1.006, *p* = 0.052) presented a *p*-value < 0.10 and were therefore selected for multivariable analysis. The selection of three variables leads to a event-per-variable ratio of about 10, which is considered sufficient to prevent analytical bias and model overfitting [21]. Multivariable regression analysis revealed that a history of previous contralateral pneumothorax was the only independent predictive factor of pneumothorax recurrence after surgery (HR 2.16, 95% C.I. 1.001–4.662, *p* = 0.049).

## 4. Discussion

Although primary spontaneous pneumothorax is considered a benign condition, it causes significant morbidity due to its high rate of recurrent episodes characterized by unclear mechanisms and risk factors [4,22]. We aimed to identify the risk factors for ipsilateral recurrence of pneumothorax after surgical treatment.

The recurrence rate found in this study (12.8%) is in line with those reported in the literature, ranging between 6.6 and 23% [23,24]. Primary spontaneous pneumothorax is a common disease, especially in young males with a low BMI value. Several studies have reported low BMI as a significant independent risk factor for recurrence after surgical treatment for PSP, suggesting that the typical morphologic habitus associated with this condition may reflect an intrinsic pleura fragility predisposing to further events [13]. In contrast with these observations, including those summarized in the recent meta-analysis by Huang et al., our results did not demonstrate a statistically significant association between BMI and post-operative recurrence, although patients in the relapse-group tended to present slightly lower BMI values [13]. Besides the constitutional characteristics associated with PSP, several exposure factors are demonstrated to play a relevant role in the predisposition to this disease: tobacco consumption increases the risk of developing a PSP approximately 9-fold in women and 22-fold in men [25,26]. However, its role as a determinant of PSP recurrence is controversial and, in accordance with other studies, our investigation did not reveal cigarettes smoking to be a significant risk factor for post-operative recurrences [27,28,29,30,31]. This finding is consistent with several previous surgical series that failed to demonstrate a clear association between smoking status and post-operative recurrence after VATS for PSP, despite the well-established role of tobacco exposure in the pathogenesis of primary spontaneous pneumothorax. For instance, Cheng et al. reported that smoking history did not significantly influence post-operative recurrence following thoracoscopic bullectomy and pleurodesis, while similar results were observed in other retrospective analyses evaluating long-term outcomes after VATS [4,28]. This discrepancy suggests that smoking may play a more relevant role in the development of a first episode of PSP rather than in determining the risk of recurrence once surgical pleurodesis has been performed.

On the other hand, our study demonstrated that a history of contralateral episodes is a significant risk factor for post-operative PSP recurrence. Our findings seem to confirm the results of a recent meta-analysis of Huang and colleagues on 23,531 patients, wherein a history of contralateral pneumothorax resulted as an independent risk factor for recurrent PSP after VATS [32]. Furthermore, several other publications pointed out that previous contralateral events of PSP represent a risk factor for subsequent post-operative relapses [32,33,34,35,36]. However, earlier surgical series reported less consistent findings. Indeed, Casali and Walker as well as Olesen et al. did not observe a statistically significant association between contralateral pneumothorax and post-operative recurrence in their multivariable analyses [4,15]. These discrepancies likely reflect differences in study design, population characteristics and surgical techniques, and highlight the persistent heterogeneity of the available evidence regarding clinical predictors of recurrence after PSP surgery. The physiopathologic mechanism behind this correlation remains uncertain; it has been hypothesized that the development of new onset subpleural blebs, bilateral PSP and relapses may be more likely in these patients due to underlying genetic or environmental factors [36]. Indeed, a history of recurrent and bilateral pneumothorax may reflect a constitutional pleural fragility that predisposes to the development of new subpleural blebs and rupture [37]. Moreover, several reports identified the presence of contralateral bullae on CT scan images as a significant risk factor for contralateral PNX development with a calculated absolute risk of 25.8–26.7%, and an increased risk of rupture as the size and number of contralateral bullae increases [22,33,34,38]. Based on these results, it has been proposed to simultaneously operate both sides when bilateral bullae were identified at CT scan images [38,39]. Indeed, it was noted that one-stage bilateral VATS reduced the risk of contralateral recurrence, with a reported rate of 2.9% and no additional surgical risk [34]. Thus, evidence of contralateral bullae in CT scans, particularly in underweight patients, has been proposed as an indication for single-stage bilateral surgery [40,41]. However, in our opinion, considering a contralateral recurrence rate of only 14.6% in PSP surgical patients with contralateral bullae, the indication for synchronous bilateral surgery is quite debatable [34,38]. On the other hand, we agree on the fact that the risk/benefit ratio of prophylactic surgery might be sufficiently high to represent a viable option in cases of high-risk occupations or activities such as scuba diving, high-altitude workers (pilots, skydivers), or workers in remote locations [39]. However, the issue has been very little debated, and further studies are needed to investigate the benefits, risks, and timing of a prophylactic treatment [35].

Interestingly, according to our results, the risk of pneumothorax recurrence appears to be associated with the size of the chest drainage positioned at the end of surgery, with larger drains representing a protective factor for recurrence. There is no consensus on this matter and the few studies focusing on the acute management of pneumothorax rather than on post-operative recurrences reported conflicting results when comparing large- and small-bore intercostal catheters in the acute management of this condition [42,43,44]. In our study, although chest drain size showed an almost significant association with recurrence in the univariate analysis, this variable did not maintain significance in the multivariable model, suggesting that the observed association may be influenced by confounding factors related to patient characteristics or intraoperative findings rather than representing an independent predictor of recurrence. It has been postulated that larger drains would allow a greater air flow and less blockage, thus favoring the obliteration of the pleural space; however, this theory would seem rational if there is clear evidence of inappropriate air drainage [44,45]. With regard to this point, a prolonged air leak and the presence of a pleural apical room following surgery demonstrated their role as risk factors for recurrence of pneumothorax in several previous experiences [23,30]. This phenomenon is thought to be determined by missed identification of air-leak points during surgery, or unsatisfactory healing of the suture line, which in turn cause an air collection within the pleural cavity, an event that radiologically may be witnessed by the presence of a residual apical space (RAS) on CXR after drain removal [30,31,46,47]. It has been assumed that, under this circumstance, lung adhesion and pleural symphysis cannot occur, leading to inadequate pleurodesis [23,48]. However, in our study we did not confirm this phenomenon: albeit a fairly high incidence of both post-operative prolonged air leak and residual apical space were recorded, we did not observe any significant correlation with recurrence of pneumothorax.

This study has several limitations that should pose some caution in interpreting our results: the retrospective nature of this study, the single-center setting and the long study period offer several biases that might affect the reliability of the conclusions. Moreover, the low number of patients and the relative low incidence of recurrences may have influenced the significance of the main analyses and the relative outcomes.

In addition, even though we have differentiated the kind of pleurodesis and bullectomy, the wide range of years considered in our analysis implies the involvement of different surgeons, various surgical devices and some minor changes in surgical techniques which were not possible to consider, due to the retrospective nature of the study. However, the institutional indications for surgical management of pneumothorax remained largely consistent, with surgery primarily indicated for recurrent pneumothorax, persistent air leak, or specific high-risk patient profiles. Regarding surgical technique, the standard approach during the entire period was video-assisted thoracoscopic surgery (VATS), while minor technical refinements occurred over time. Similarly, perioperative management, post-operative care protocols, did not undergo substantial institutional changes that would meaningfully influence the outcomes analyzed. Nevertheless, we acknowledge that some minor unmeasured changes in routine clinical practice during the time interval may have occurred and influenced the results of our analysis. Eventually, since patients were not posed under a regular radiological follow-up and recurrences were established in case of suggestive symptoms and subsequent radiological confirmation, we cannot exclude that asymptomatic or mildly symptomatic events may have occurred. Therefore, the number of events might have been underestimated.

## 5. Conclusions

In conclusion, the presence of a positive history of previous episodes of pneumothorax, notably contralateral to the site of surgery, is an important risk factor for the development of post-operative recurrence. This anamnestic profile could be considered in defining surgery indications and procedure strategies to be adopted, contemplating multiple and more radical techniques of pleurodesis to prevent future episodes in this delicate subset of patients.

## Figures and Tables

**Table 1:** Population characteristics.

	All 226	Recurrence 29 (12.8)	No Recurrence 197 (87.2)	*p*
Age	22 (IQR 18–30)	21 (IQR 18–25)	22 (IQR 18–31)	0.329
Male Sex	182 (80.5)	21 (72.4)	161 (81.7)	0.313
BMI (kg/m^2^)	19.8 (IQR 18.4–22.2)	19.3 (IQR 17.5–20.8)	20.0 (IQR 18.4–22.2)	0.094
Smoke	102 (45.1)	13 (44.8)	89 (45.2)	1.000
Comorbidities	62 (27.4)	5 (17.2)	57 (28.9)	0.265
Pulmonary	14 (22.6)	0 (0)	14 (7.1)	0.225
Cardiac	8 (12.9)	1 (3.4)	7 (3.6)	1.000
Renal	3 (4.8)	1 (3.4)	2 (1)	0.339
Hepatic	2 (3.2)	0 (0)	2 (1)	1.000
Other	45 (72.6)	3 (10.3)	42 (21.3)	0.217

Data reported as number (%), mean ± sd or median (IQR). Abbreviations: BMI: body mass index.

**Table 2:** Presentation of pneumothorax.

	All 226	Recurrence 29 (12.8)	No Recurrence 197 (87.2)	*p*
Side				0.109
Right	131 (58.0)	21 (72.4)	110 (55.8)	
Left	95 (42.0)	8 (27.6)	87 (44.2)	
Size of PNX (cm)				
Apical *				0.359
≤3	61 (30.6)	5 (18.5)	56 (32.6)	
3–6	63 (31.7)	10 (37.0)	53 (30.8)	
>6	75 (37.7)	12 (44.5)	63 (36.6)	
Hylum **				0.758
≤2	98 (50.8)	13 (48.1)	85 (51.2)	
2–4	36 (18.7)	4 (14.8)	32 (19.3)	
>4	59 (30.5)	10 (37.1)	49 (29.5)	
Effusion	78 (34.5)	10 (34.5)	68 (34.5)	1.000
CT	73 (32.3)	7 (24.1)	66 (33.5)	
Bullae at CT	42 (57.5)	2 (28.6)	40 (60.6)	0.127
Previous Episodes	154 (68.1)	24 (82.8)	130 (66.0)	0.079
Number of Episodes				0.149
1	108 (71.1)	14 (60.9)	94 (72.9)	
2	30 (19.7)	5 (21.7)	25 (19.4)	
3	12 (7.9)	4 (17.4)	8 (6.1)	
4	2 (1.3)	0 (0)	2 (1.6)	
Ipsilateral to Site of Surgery	124 (54.9)	18 (62.1)	106 (53.8)	0.431
Contralateral to Site of Surgery	49 (21.7)	11 (37.9)	38 (19.3)	0.030
Thoracostomy Before Surgery	158 (69.9)	22 (75.9)	136 (69.0)	0.522
PNX-Surgery Interval (days)	4 (IQR 2–7)	3 (IQR 2–6)	5 (IQR 2–7)	0.247

Data reported as number (%), mean ± sd or median (IQR). Abbreviations: PNX: pneumothorax; CT: computed tomography; Ch: Charrière. * Data available on 199 patients. ** Data available on 193 patients.

**Table 3:** Indication for surgery and operative data.

	All 226	Recurrence 29 (12.8)	No Recurrence 197 (87.2)	*p*
Indication to Surgery				0.875
Ipsilateral Recurrence	117 (51.8)	16 (55.2)	100 (50.8)	
Persistent Air Leak	71 (31.4)	8 (27.6)	65 (33)	
Contralateral Recurrence	26 (11.5)	4 (13.8)	21 (10.6)	
Other	12 (5.3)	1 (3.4)	11 (5.6)	
Surgical Technique				0.566
VATS	220 (97.3)	28 (96.6)	192 (97.5)	
Bullae Resection	197 (87.2)	26 (89.7)	171 (86.8)	1.000
Stapler	178 (90.4)	23 (88.5)	155 (90.6)	
Ligation	19 (9.6)	3 (11.5)	16 (9.4)	
Pleurodesis	224 (99.1)	29 (100)	195 (99)	1.000
Surgical Pleurodesis	223 (98.7)	29 (100)	194 (98.5)	1.000
Pleurectomy	109 (48.2)	14 (48.3)	95 (48.2)	1.000
Abrasion/Scarification/None	117 (51.8)	15 (51.2)	102 (51.8)	
Medical Pleurodesis	29 (12.8)	4 (13.8)	25 (12.7)	0.773
Time of Surgery (min)	65 (IQR 55–86.5)	60 (IQR 45–85)	70 (IQR 50–90)	0.271
Number of Chest Drains				0.462
1	180 (79.6)	25 (86.2)	155 (78.7)	
2	46 (20.4)	4 (13.8)	42 (21.3)	
Largest Chest Drain Caliber				0.032
20–24 Ch	84 (37.2)	16 (55.2)	68 (34.5)	
28–32 Ch	142 (62.8)	13 (44.8)	129 (65.5)	

Data reported as number (%), mean ± sd or median (IQR). Abbreviations: VATS: video-assisted thoracoscopic surgery; Ch: Charrière.

**Table 4:** Post-operative results.

	All 226	Recurrence 29 (12.8)	No Recurrence 197 (87.2)	*p*
First Day Apical Room	119 (52.7)	16 (55.2)	103 (52.3)	0.843
PNX Size	1 (IQR 0.5–2)	0.9 (IQR 0.6–1.7)	1 (IQR 0.5–2)	0.563
Post-operative Air Leak	111 (49.1)	14 (48.3)	97 (49.2)	1.000
Drains Removal (days)	4 (IQR 4 -6)	4 (IQR 4–5)	4 (IQR 4–6)	0.542
Post-removal Apical Room	144 (63.7)	19 (65.5)	125 (63.5)	1.000
Length of Stay (days)	5 (IQR 4–7)	5 (IQR 0–7)	5 (IQR 0–7)	0.743
Complications	54 (23.9)	8 (27.6)	46 (23.4)	0.643
Bleeding	6 (2.3)	1 (3.4)	5 (2.5)	0.566
Persistent Air Leak	22 (11.9)	4 (13.8)	18 (9.1)	0.498
Pneumonia	1 (0.4)	0 (0)	1 (0.5)	1.000
Fever	12 (5.3)	0 (0)	12 (6.1)	0.147
Chronic Pain	3 (1.3)	0 (0)	3 (1.5)	1.000
Other	9 (3.9)	2 (6.9)	7 (3.6)	0.325

Data reported as number (%), mean ± sd or median (IQR). Abbreviations: PNX: pneumothorax.

**Table 5:** Cox regression analysis for PNX recurrence.

		Univariable				Multivariable			
		HR	95% C.I.	*p*		HR	95% C.I.	*p*	
Age		0.98	0.936–1.016	0.233					
BMI		0.87	0.748–0.999	0.049	*	0.89	0.759–1.031	0.127	
Sex	F vs. M	0.28	0.696–3.556	0.276					
Smoking	Yes vs. No	0.98	0.469–2.029	0.947					
Previous Omolateral Episode	Yes vs. No	1.29	0.611–2.742	0.500					
Previous Contralateral Episode	Yes vs. No	2.68	1.266–5.680	0.010	*	2.16	1.001–4.662	0.049	*
Surgical Pleurodesis	Yes vs. No	1.09	0.527–0.527	0.809					
Medical Pleurodesis	Yes vs. No	0.93	0.321–2.677	0.889					
Post-op Air Leak	Yes vs. No	1.04	0.503–2.165	0.909					
Apical Room After Drain Removal	Yes vs. No	1.15	0.532–2.480	0.724					
Persistent Air Leak	Yes vs. No	1.61	0.56–4.629	0.377					
Largest Chest Drain	28–32 vs. 20–24	0.48	0.232–1.006	0.052		0.59	0.284–1.263	0.173	

* significant *p*-value.

## Data Availability

The raw data supporting the conclusions of this article will be made available by the authors upon reasonable request.

## Abbreviations

The following abbreviations are used in this manuscript:

BMI	Body Mass Index
Ch	Charriere
CT	Computed Tomography
CXR	Chest X-ray
PNX	Pneumothorax
PSP	Primary Spontaneous Pneumothorax
STROBE	Strengthening the Reporting of Observational Studies in Epidemiology
VATS	Video-Assisted Thoracic Surgery

## References

[1] Baumann M.H. (2001). Pneumothorax. Semin. Respir. Crit. Care Med..

[2] Amer K. (2019). Pneumothorax.

[3] Baumann M.H., Noppen M. (2004). Pneumothorax. Respirology.

[4] Olesen W.H., Lindahl-Jacobsen R., Katballe N., Sindby J.E., Titlestad I.L., Andersen P.E., Licht P.B. (2016). Recurrent Primary Spontaneous Pneumothorax is Common Following Chest Tube and Conservative Treatment. World J. Surg..

[5] Kepka S., Dalphin J.C., Parmentier A.L., Pretalli J.B., Gantelet M., Bernard N., Mauny F., Desmettre T. (2017). Primary Spontaneous Pneumothorax Admitted in Emergency Unit: Does First Episode Differ from Recurrence? A Cross-Sectional Study. Can. Respir. J..

[6] Schramel F.M., Sutedja T.G., Braber J.C., van Mourik J.C., Postmus P.E. (1996). Cost-effectiveness of video-assisted thoracoscopic surgery versus conservative treatment for first time or recurrent spontaneous pneumothorax. Eur. Respir. J..

[7] Torresini G., Vaccarili M., Divisi D., Crisci R. (2001). Is video-assisted thoracic surgery justified at first spontaneous pneumothorax?. Eur. J. Cardiothorac. Surg..

[8] Plojoux J., Froudarakis M., Janssens J.P., Soccal P.M., Tschopp J.M. (2019). New insights and improved strategies for the management of primary spontaneous pneumothorax. Clin. Respir. J..

[9] Desai U.D., Karkhanis V., Joshi J.M. (2019). Pneumothorax. Pneumon.

[10] Wong A., Galiabovitch E., Bhagwat K. (2019). Management of primary spontaneous pneumothorax: A review. ANZ J. Surg..

[11] Chambers A., Scarci M. (2009). In patients with first-episode primary spontaneous pneumothorax is video-assisted thoracoscopic surgery superior to tube thoracostomy alone in terms of time to resolution of pneumothorax and incidence of recurrence?. Interact. Cardiovasc. Thorac. Surg..

[12] Pogorelić Z., Gudelj R., Bjelanović D., Jukić M., Elezović Baloević S., Glumac S., Furlan D. (2020). Management of the Pediatric Spontaneous Pneumothorax: The Role of Video-Assisted Thoracoscopic Surgery. J. Laparoendosc. Adv. Surg. Tech. A.

[13] Huang H., Ji H., Tian H. (2015). Risk factors for recurrence of primary spontaneous pneumothorax after thoracoscopic surgery. Biosci. Trends.

[14] Riveiro-Blanco V., Pou-Álvarez C., Ferreiro L., Toubes M.E., Quiroga-Martínez J., Suárez-Antelo J., García-Prim J.M., Rivo-Vázquez J.E., Castro-Calvo R., González-Barcala F.J. (2022). Recurrence of primary spontaneous pneumothorax: Associated factors. Pulmonology.

[15] Walker S.P., Bibby A.C., Halford P., Stadon L., White P., Maskell N.A. (2018). Recurrence rates in primary spontaneous pneumothorax: A systematic review and meta-analysis. Eur. Respir. J..

[16] Vandenbroucke J.P., von Elm E., Altman D.G., Gøtzsche P.C., Mulrow C.D., Pocock S.J., Poole C., Schlesselman J.J., Egger M. (2007). Strengthening the Reporting of Observational Studies in Epidemiology (STROBE): Explanation and elaboration. Epidemiology.

[17] Società Italiana di Chirurgia Toracica (2022). Pneumotorace Spontaneo Primitivo. Linee Guida della Società Italiana di Chirurgia Toracica. https://www.iss.it/-/snlg-pneumotorace-spontaneo-primitivo.

[18] Woo W., Kim C.H., Kim B.J., Song S.H., Moon D.H., Kang D.Y., Lee S. (2021). Early Postoperative Pneumothorax Might Not Be ‘True’ Recurrence. J. Clin. Med..

[19] Brophy S., Brennan K., French D. (2021). Recurrence of primary spontaneous pneumothorax following bullectomy with pleurodesis or pleurectomy: A retrospective analysis. J. Thorac. Dis..

[20] Onuki T., Ueda S., Yamaoka M., Sekiya Y., Yamada H., Kawakami N., Araki Y., Wakai Y., Saito K., Inagaki M. (2017). Primary and Secondary Spontaneous Pneumothorax: Prevalence, Clinical Features, and In-Hospital Mortality. Can. Respir. J..

[21] Vittinghoff E., McCulloch C.E. (2007). Relaxing the rule of ten events per variable in logistic and Cox regression. Am. J. Epidemiol..

[22] Nakayama T., Takahashi Y., Uehara H., Matsutani N., Kawamura M. (2017). Outcome and risk factors of recurrence after thoracoscopic bullectomy in young adults with primary spontaneous pneumothorax. Surg. Today.

[23] Gaunt A., Martin-Ucar A.E., Beggs L., Beggs D., Black E.A., Duffy J.P. (2008). Residual apical space following surgery for pneumothorax increases the risk of recurrence. Eur. J. Cardiothorac. Surg..

[24] Bialas R.C., Weiner T.M., Phillips J.D. (2008). Video-assisted thoracic surgery for primary spontaneous pneumothorax in children: Is there an optimal technique?. J. Pediatr. Surg..

[25] Bense L., Eklund G., Wiman L.G. (1987). Smoking and the increased risk of contracting spontaneous pneumothorax. Chest.

[26] Wallaert B., Gressier B., Marquette C.H., Gosset P., Remy-Jardin M., Mizon J., Tonnel A.B. (1993). Inactivation of alpha 1-proteinase inhibitor by alveolar inflammatory cells from smoking patients with or without emphysema. Am. Rev. Respir. Dis..

[27] Lippert H.L., Lund O., Blegvad S., Larsen H.V. (1991). Independent risk factors for cumulative recurrence rate after first spontaneous pneumothorax. Eur. Respir. J..

[28] Cheng Y.L., Huang T.W., Lin C.K., Lee S.C., Tzao C., Chen J.C., Chang H. (2009). The impact of smoking in primary spontaneous pneumothorax. J. Thorac. Cardiovasc. Surg..

[29] Uramoto H., Shimokawa H., Tanaka F. (2012). What factors predict recurrence of a spontaneous pneumothorax?. J. Cardiothorac. Surg..

[30] Imperatori A., Rotolo N., Spagnoletti M., Festi L., Berizzi F., Di Natale D., Nardecchia E., Dominioni L. (2015). Risk factors for postoperative recurrence of spontaneous pneumothorax treated by video-assisted thoracoscopic surgery. Interact. Cardiovasc. Thorac. Surg..

[31] Cattoni M., Rotolo N., Mastromarino M.G., Cardillo G., Nosotti M., Mendogni P., Rizzi A., Raveglia F., Siciliani A., Rendina E.A. (2020). Analysis of pneumothorax recurrence risk factors in 843 patients who underwent videothoracoscopy for primary spontaneous pneumothorax: Results of a multicentric study. Interact. Cardiovasc. Thorac. Surg..

[32] Huang N., He S., Chen S., Zhang G., Ruan L., Huang J. (2024). Incidence and risk factors for recurrent primary spontaneous pneumothorax after video-assisted thoracoscopic surgery: A systematic review and meta-analysis. J. Thorac. Dis..

[33] Jang H.J., Lee J.H., Nam S.H., Ro S.K. (2020). Fate of contralateral asymptomatic bullae in patients with primary spontaneous pneumothorax. Eur. J. Cardiothorac. Surg..

[34] Liu Y.W., Chang P.C., Chang S.J., Chiang H.H., Li H.P., Chou S.H. (2020). Simultaneous bilateral thoracoscopic blebs excision reduces contralateral recurrence in patients undergoing operation for ipsilateral primary spontaneous pneumothorax. J. Thorac. Cardiovasc. Surg..

[35] Noh D., Keum D.Y., Park C.K. (2015). Outcomes of Contralateral Bullae in Primary Spontaneous Pneumothorax. Korean J. Thorac. Cardiovasc. Surg..

[36] Onuki T., Kawamura T., Kawabata S., Yamaoka M., Inagaki M. (2019). Neo-generation of neogenetic bullae after surgery for spontaneous pneumothorax in young adults: A prospective study. J. Cardiothorac. Surg..

[37] Shigenobu T., Ohtsuka T., Yoshizu A. (2023). Risk factors for the recurrence of primary spontaneous pneumothorax after video-assisted thoracoscopic surgery in patients younger than 40 years. J. Thorac. Dis..

[38] Jeong J.Y., Shin A.Y., Ha J.H., Suh J.H., Choi S.Y., Kim J.S., Park C.B. (2022). Natural History of Contralateral Bullae/Blebs After Ipsilateral Video-Assisted Thoracoscopic Surgery for Primary Spontaneous Pneumothorax: A Retrospective Cohort Study. Chest.

[39] Kennedy N., Petrakis N., Chan J., Jurisevic C. (2023). Spontaneous pneumothorax rates following video-assisted thoracoscopic talc pleurodesis with or without resection of macroscopic bullous disease. ANZ J. Surg..

[40] Chou S.H., Li H.P., Lee J.Y., Chang S.J., Lee Y.L., Chang Y.T., Kao E.L., Dai Z.K., Huang M.F. (2010). Is prophylactic treatment of contralateral blebs in patients with primary spontaneous pneumothorax indicated?. J. Thorac. Cardiovasc. Surg..

[41] Huang T.W., Lee S.C., Cheng Y.L., Tzao C., Hsu H.H., Chang H., Chen J.C. (2007). Contralateral recurrence of primary spontaneous pneumothorax. Chest.

[42] Vedam H., Barnes D.J. (2003). Comparison of large- and small-bore intercostal catheters in the management of spontaneous pneumothorax. Intern. Med. J..

[43] Tsai W.K., Chen W., Lee J.C., Cheng W.E., Chen C.H., Hsu W.H., Shih C.M. (2006). Pigtail catheters vs large-bore chest tubes for management of secondary spontaneous pneumothoraces in adults. Am. J. Emerg. Med..

[44] McCracken D., Psallidas I., Rahman N. (2018). Chest drain size: Does it matter?. Eurasian J. Pulmonol..

[45] Hallifax R.J., Psallidas I., Rahman N.M. (2017). Chest Drain Size: The Debate Continues. Curr. Pulmonol. Rep..

[46] How C.H., Tsai T.M., Kuo S.W., Huang P.M., Hsu H.H., Lee J.M., Chen J.S., Lai H.S. (2014). Chemical pleurodesis for prolonged postoperative air leak in primary spontaneous pneumothorax. J. Formos. Med. Assoc..

[47] Gómez-Caro A., Moradiellos F.J., Larrú E., Díaz-Hellín V., Marrón C., Pérez-Antón J.A., Martín de Nicolás J.L. (2006). Effectiveness and complications of video-assisted surgery for primary spontaneous pneumothorax. Arch. Bronconeumol..

[48] Waller D.A., Forty J., Morritt G.N. (1994). Video-assisted thoracoscopic surgery versus thoracotomy for spontaneous pneumothorax. Ann. Thorac. Surg..

